# The Royal Commission on Tuberculosis

**Published:** 1895-05-04

**Authors:** 


### MEDICAL PROGRESS AND HOSPITAL CLINICS.

[*The Editor will be glad to receive offers of co-operation and contributions from members of the profession. All letters should be addressed to* THE EDITOR, THE LODGE, PORCHESTER SQUARE, LONDON, W.]

### THE ROYAL COMMISSION ON TUBERCULOSIS.

The report of the Royal Commission appointed nearly five years ago to inquire into the effect of food derived from tuberculosis animals on human health has been presented to Parliament. Underlying the whole subject of the communicability of tuberculosis is the question whether or no the disease as it appears in animals and in man is the same, and whether the different forms in which it shows itself in man, viz., consumption of the lung, consumption of the bowel, general tuberculosis, and that localised tuberculosis which goes by the name of scrofula, are merely different modes of development of the same disease. Both these questions the Commissioners answer in the affirmative. The course is thus cleared for the investigation of the subject in hand. It is accepted as capable of demonstration that, however different tuberculosis in animals may appear from tuberculosis in man, it is in each case " the same disease," and that the varied manifestations of the disease in man are due to variations of environment, but not to any difference in the nature of the infection; and as clenching this it is pointed out that the results of the investigation show that tuberculous disease in man is the same, whether it is derived from food or from other sources.

After saying so much it is obvious that the result of the inquiry has been to prove that the infection of tuberculosis can be received by way of food; and, knowing as we do the frequency with which tuberculosis is found among animals destined for human consumption, and especially in milk which is so often taken raw, we naturally inquire what are the circumstances which favour or retard this transmission of disease by food?

The experiments of Dr. Sidney Martin and Dr. Sims Woodhead prove the extreme danger of eating tuberculous material in the raw state. Each of Dr. Martin's animals received only a single dose; one pig, eight guinea pigs, and ten calves each received a dose, and of them the pig, six guinea-pigs, and eight calves became tuberculous. In Dr. Woodhead's experiments tuberculous disease followed feeding on uncooked tuberculous matter in all the pigs (seven in number), in all the cats (five), and in fifty guinea-pigs out of seventy-six.

It is important to contrast this great mortality after eating material known to contain tubercle, with the result of feeding on "meat" in the ordinary, or butcher's acceptation of the term, *i.e.*, muscle, &c., taken from tuberculous animals, but free from obvious masses of tubercle, or on milk taken from tuberculous cows whose udders were not diseased. Of these, 36 per cent. of the pigs, 16 per cent. of the guinea-pigs, and 15 per cent. of the rabbits became tuberculous. None of the control animals were affected.

These experiments clearly show that the bodies of tuberculous animals are not infectious in a uniform degree throughout, and that the danger especially resides in the portions actually affected with tuberculosis.

Further investigation shows that these portions are chiefly the glands and organs which are usually cut away by the butcher, so that "in muscle and muscle juice it is very seldom that tubercle bacilli are to be met with," even where the organs exhibit very advanced or generalised tuberculosis.

On the other hand, it is very difficult to make sure of the absence of tuberculous matter from any part of a carcass that shows evidence of tubercle elsewhere, and it was proved by inoculation experiments that tubercle was present in some cases which failed to react to the rougher test of feeding. Moreover, it seems that there is always the danger of good meat being smeared with tuberculous matter by means of the knives or hands or cloths of the butchers, if in the same abattoir tuberculous animals are being slaughtered; and Dr. Martin says, " One is driven to the conclusion that, when meat is infective, it commonly acquires its properties by being accidentally contaminated with tuberculous material during its removal from the carcass." This is of great importance, for it becomes clear that it is not enough to exclude tuberculous meat from the butcher's stall—tuberculous animals must, as far as possible, be excluded from the slaughter-house; while, on the other hand, a great deal of the meat even of tuberculous animals would be available for food if proper care were exercised in dealing with it.

The teaching to be derived from this is that not only must the animals and the carcasses be inspected, but that a careful and continuous inspection must be exercised over the whole process and machinery of slaughtering and dealing with the meat. The bearing of this upon the "dead meat" trade must not be forgotten, for the Commissioners point out that "little evidence about the more serious degrees of tuberculosis would be discoverable in carcasses from which the organs had been removed; and that this is habitually the case with so-called 'dead meat,' whether English or foreign."

In regard to milk, the extremely important observation has been made that unless the udder be affected with disease the milk even of tuberculous cows remains free from infective properties. Whenever, however, there is tuberculous disease of the udder the milk is virulently infectious. Hence, in view of the rapidity with which tuberculosis of the udder may become developed, it must be admitted that the presence of a tuberculous cow in a dairy is a decided source of danger to the public.

The one bit of comfort we can derive from the Report is as to the influence of cooking on such bacilli as may be contained in milk or smeared on the outside of joints of meat.

Dr. Woodhead says: "The most deadly tuberculous material can be rendered absolutely innocuous, so far as any spreading infectious disease is concerned, by the action of a temperature at which water boils."

Hence the recommendation by the Commissioners of "the simple expedient of putting every suspected milk over the fire, and taking it off when it boils." And, as things are at present, what milk should not be suspected?

In regard to meat also, cooking destroys the harmful properties of tuberculous matter, so far as the required heat penetrates. If the joints are not rolled up, the most dangerous part, *i.e.*, the outside, is usually disinfected by ordinary cooking. On the other hand, ordinary cooking cannot be relied on in the slightest degree to sterilise tuberculous material included in the centre of rolls of meat, especially if these are more than three pounds or four pounds weight. The least reliable method of cooking for the purpose is roasting before the fire, next comes roasting in an oven, and then boiling.

The whole report is one of very great interest, and no less interesting is the question, What action will be taken upon it?

